# The association between serum uric acid and depression among U.S. National Health and Nutrition Examination Survey

**DOI:** 10.3389/fnut.2025.1517744

**Published:** 2025-04-08

**Authors:** Hong He, Ping Li, Haokun Huang, Yanlin Zeng, Min Zhang, Zhibing Chen, Shiqi Huang, Fangfang Zeng, Hui Ge

**Affiliations:** ^1^Healthcare Outpatient Center, The First Affiliated Hospital of Sun Yat-sen University, Guangzhou, China; ^2^Department of Plastic Surgery, The First Affiliated Hospital of Guangdong Pharmaceutical University, Guangzhou, China; ^3^Department of Public Health and Preventive Medicine, School of Medicine, Jinan University, Guangzhou, China; ^4^School of Public Health, Sun Yat-sen University, Guangzhou, China

**Keywords:** uric acid, depression, subgroup analyses, a cross-sectional analysis, NHANES

## Abstract

**Background:**

Previous studies have suggested that serum uric acid (UA) may influence depression through oxidative stress pathways, but with inconclusive results. This study aimed to investigate the association between serum UA levels and depression, as well as potential variations across demographic subgroups in the U.S. adult population.

**Methods:**

A cross-sectional analysis of 23,059 participants from NHANES (2013–2018) was conducted. Serum UA levels were classified into quartiles, and depression was measured using the Patient Health Questionnaire-9 (PHQ-9), with a score ≥ 10 indicating depression. Weighted logistic regression models were used to analyze the association between UA levels and depression, adjusting for demographic and clinical covariates. Subgroup analyses were performed to assess variations by sex, age, race/ethnicity, and other health-related factors.

**Results:**

Participants in the highest UA quartile had a lower prevalence of depression compared to those in the lowest quartile (13.9% vs. 16.2%, *p* = 0.004). After adjusting for covariates, higher UA levels were significantly associated with a reduced risk of depression (OR = 0.76, 95% CI: 0.67, 0.86, *p* < 0.001 for quartile 4). Subgroup analyses indicated significant interactions by age (p for interaction = 0.051), race/ethnicity (p for interaction = 0.027), and history of cardiovascular disease (p for interaction = 0.005), with more pronounced inverse associations observed among older adults, other races and participants with cardiovascular disease.

**Conclusion:**

Higher serum UA levels were inversely associated with depression among U.S. adults, especially among races, participants with cardiovascular disease and older age. Further research is needed to confirm these findings and investigate potential underlying mechanisms.

## 1 Introduction

According to the World Health Organization (WHO), depressive disorder has become a common mental disorder, affecting 280 million people in the world, including 5% of adults and 5.7% of adults older than 60 years (1). In particular, depression has been known as one of the key risk factors of suicide, which causes more than 700,000 people to die and is the fourth leading cause of death among adults across the world (2, 3). Thus, there is a pressing need for research into modifiable risk factors for depression.

The exact pathophysiological pathogenesis of depression is still unclear, but the increased vulnerability to depressive-like behaviors is associated with impaired oxidative stress balance (4). Oxidative stress leads to reduced brain neurogenesis and increased neuronal apoptosis, while also disrupting 5-HT neurotransmitter activity and monoamine neurotransmitter metabolism (5). In patients with depression, oxidative stress is increased, whereas total antioxidant capacity is decreased, contributing to various depressive symptoms (6). Exploring factors related to oxidative stress may offer potential strategies for preventing and treating depression.

Many factors influence oxidative stress, including social, psychological, and biological factors, and lifestyle (physical inactivity or harmful use of alcohol) (1). Serum uric acid (UA), as the ultimate product of decomposing the body’s purine compounds, is a potent radical scavenger in plasma (7). Specially, as the soluble form of UA, the serum urate acts as a protective powerful antioxidant under physiological concentrations (7), accounting for over 60% of antioxidant activity in plasma (8). Its mechanisms include maintaining peroxidase activity, preventing the formation of harmful free radicals like superoxide and peroxynitrite, protecting cellular enzymes from oxidative damage, and inhibiting iron-dependent oxidative reactions (9, 10). As part of the non-enzymatic antioxidant systems, it is possible to speculate that the serum UA may also be involved in the pathophysiological pathways of depression through oxidative stress and other mechanisms (4).

Prior studies have suggested that elevated serum urate levels were associated with decreased risks of neurodegenerative diseases, including Alzheimer’s disease (11), Parkinson’s disease (12), and multiple sclerosis (13). A meta-analysis indicated that higher UA has a deleterious impact on attention and executive function (14). Serum UA levels in patients with bipolar disorder were higher than health controls, in those with depression, or depressed episode patients (15). Several studies have explored the association between serum UA and depression across different populations. Results in a southwestern Chinese cohort identified lower SUA in depressed patients, whereas higher SUA was associated with bipolar disorder (16). Two independent cohorts linked elevated SUA levels to a reduced risk of depression-related hospitalization (17). In a Chinese study of middle-aged and elderly participants, a negative association between SUA and depression was found in men but not in women (18). Moreover, a study from Korea reported opposing associations between SUA and depressive symptoms depending on the presence of low-grade inflammation (19). Given this inconclusive evidence linking SUA to depression, the directionality of this relationship remains unclear, warranting further investigation among different populations.

Thus, this study aimed to explore association between serum UA levels and depression using data from the U.S. National Health and Nutrition Examination Survey (NHANES) 2013–2018. Additionally, we also analyzed whether this association varied across different population subgroups, including age, sex, and racial/ethnic groups.

## 2 Methods

### 2.1 Data source and study population

This cross-sectional study used the data from the National Health and Nutrition Examination Survey (NHANES) 2005–2018. NHANES is a nationally representative survey designed to assess the health and nutritional status of noninstitutionalized U.S. civilians older than 2 months every 2 years, conducted by the Centers for Disease Control and Prevention (CDC). The NHANES data are publicly accessible through the CDC website,^1^ including data on demographic, socioeconomic, dietary, physiological, laboratory information, and health outcomes. The survey protocol was approved by the National Center for Health Statistics (NCHS) Research Ethics Review Board, and the Declaration of Helsinki was followed when conducting the study. Written informed consent was obtained from all participants prior to data collection.

**Figure 1:**
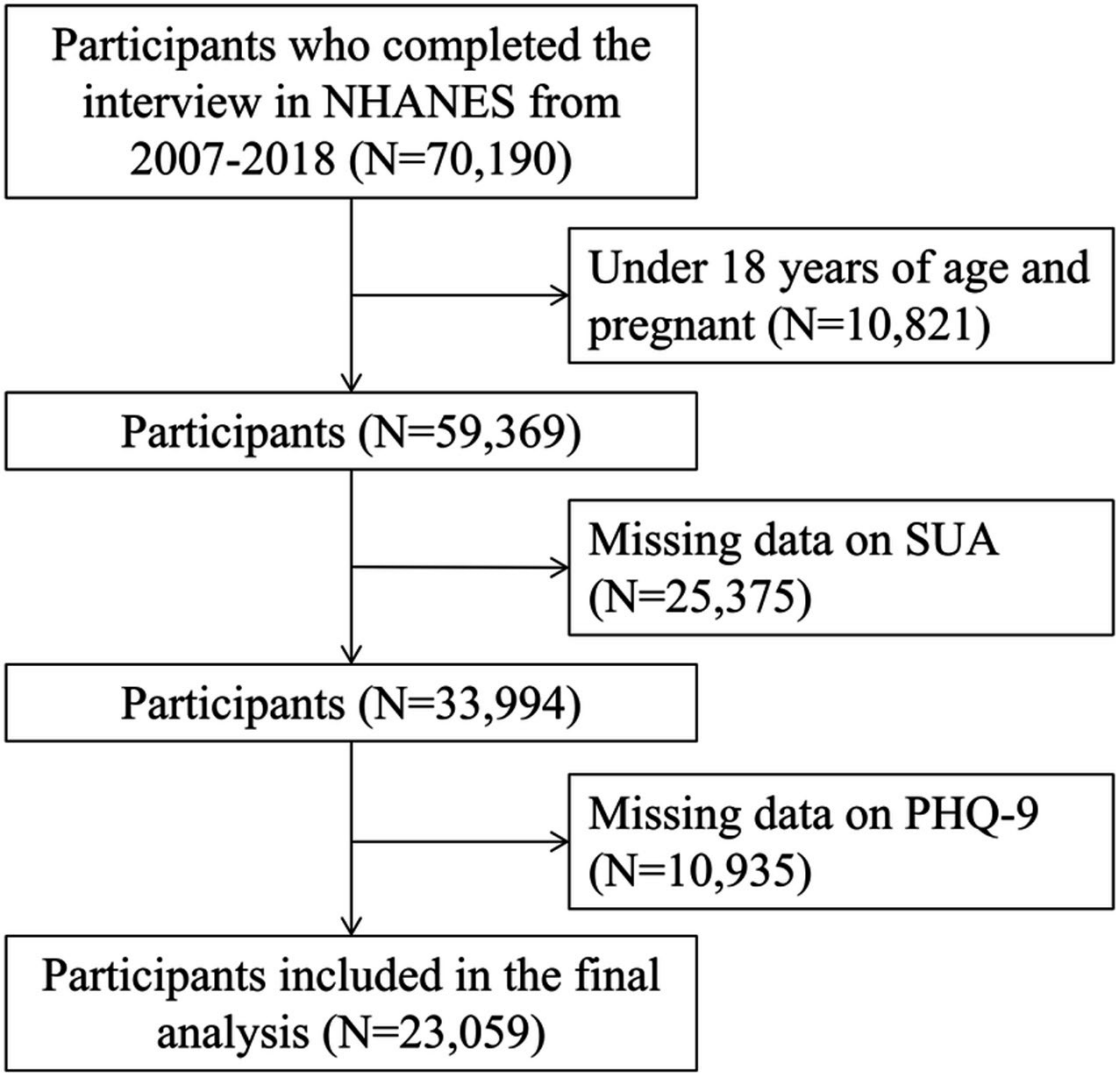
Flowchart of participants selection. NHANES, National Health and Nutrition Examination Survey; PHQ-9, Patient Health Questionnaire-9; SUA, serum uric acid.

Data from 8 consecutive NHANES cycles (2003–2004, 2005–2006, 2007–2008, 2009–2010, 2011–2012, 2013–2014, 2015–2016, and 2017–2018) were initially considered, encompassing 70,190 individuals. The participants were excluded if they had at least one of the following conditions: (1) individuals under 18 years of age (*N* = 10,568); (2) pregnant participants (*N* = 253); (2) individuals missing data on SUA levels (*N* = 25,375); and (3) participants lacking data on Patient Health Questionnaire-9 (PHQ-9) for definition of depression (*N* = 10,935), a final analytic sample of 23,059 participants was obtained. The flow chart of the screening process is shown in Figure 1.

#### 2.1.1 SUA measurement

The SUA measurement was performed by using a Beckman Synchron LX20 in the NHANES 2001–2007 (20), a Beckman UniCel DxC800 Synchron in the NHANES 2008–2016 (21), and a Roche Cobas 6,000 analyzer in the NHANES 2017–2018 (22). Values reported in milligrams per deciliter (mg/dL) were converted to micromoles per liter (μmol/L) by multiplying of 59.48. The use of SUA from multiple cycles for analysis has been presented in previous study (23).

#### 2.1.2 Assessment and definition of depression

Depression was assessed using the Patient Health Questionnaire-9 (PHQ-9), a widely validated self-report instrument designed to identify the presence and severity of depressive symptoms over the preceding 2 weeks, based on the diagnostic criteria outlined in the Diagnostic and Statistical Manual of Mental Disorders, Fourth Edition (DSM-IV) (24). The PHQ-9 questionnaire consists of nine items, each scored on a scale from 0 (“not at all”) to 3 (“almost every day”), yielding a total score range of 0–27. A higher total score reflects greater symptom severity. In this study, participants with a total PHQ-9 score ≥ 10 were classified as having depression, based on established thresholds for identifying major depressive disorder (25). This cut-off has demonstrated high diagnostic accuracy, with both sensitivity and specificity of 88% for major depression (25).

#### 2.1.3 Assessment of covariates

Covariates included in the present study were selected based on previous studies (23, 26, 27). Demographic information was obtained from interview questionnaires, including age, gender, race/ethnicity, education level, family income, smoking and drinking status. Age was categorized into three groups: <30 years, 30–59 years, and ≥ 60 years. Race/ethnicity was stratified into non-Hispanic White, non-Hispanic Black, Hispanic, and other races. Education level was divided into three categories: less than high school, high school, and college or above. Family income was classified by using the income-to-poverty ratio, categorized into <1, 1–3, and > 3, with higher values indicating better economic status. Smoking status was assessed based on participants’ lifetime history of smoking at least 100 cigarettes and categorized into never smokers, former smokers, and current smokers. Alcohol consumption was determined through 24-h dietary recall and classified as never (0 g/day), moderate (0.1–27.9 g/day for men and 0.1–13.9 g/day for women), or heavy drinking (≥28 g/day for men and ≥ 14 g/day for women).

Anthropometric measurements including height and weight were measured following the relevant standardized protocols, and BMI was calculated as weight in kilograms divided by height in meters squared. Hypertension was defined according to the 2017 American College of Cardiology/American Heart Association guidelines, as systolic blood pressure ≥ 130 mmHg, diastolic blood pressure ≥ 80 mmHg, or the use of antihypertensive medications. History of diabetes was diagnosed when one of the following conditions was met: self-reported diabetes, or fasting plasma glucose ≥ 7.0 mmoL/L, or glycated hemoglobin (HbA1c) ≥ 6.5%, or glucose ≥ 11.1 mmoL/L, or ever using insulin or diabetes medication. History of cardiovascular disease (CVD) was defined based on physician ever diagnoses of congestive heart failure or stroke. Additional clinical variables included fasting serum glycated hemoglobin (HbA1c), triglycerides, total cholesterol, and creatinine were also extracted.

### 2.2 Statistical analysis

All analyses were conducted following the NHANES analytical guidelines. To account for the complex, multistage probability sampling design of NHANES, sample weights, strata, and primary sampling units (PSUs) were incorporated into all analyses to ensure nationally representative estimates.

SUA were classified according to quartiles with quartile 1 (Q1) having the lowest level and quartile 4 (Q4) having the highest level. In this study, descriptive statistics were used to summarize demographic and clinical covariates according to quartiles of SUA. Descriptive statistics were used to summarize demographic and clinical covariates. Continuous variables were expressed as means and standard deviations, and categorical variables were presented as n (%). Comparisons for continuous variables were performed using the one-way ANOVA analysis and categorical variables were compared using the Chi-square test. For covariates with missing data, multiple imputation was performed using the Mice package in R.

Weighted logistic regression models were employed to examine the association between SUA levels and depression, adjusting for potential confounders, with the Q1 as the reference group. Three models were applied, with model 1 without adjustment. Model 2 adjusted for age, sex, and race/ethnicity. For model 3, we further adjusted smoking status, drinking status, BMI, education level, economic status, TC, TG, glycated hemoglobin, creatinine, and personal history of hypertension, diabetes, and CVD. Odds ratios (OR) and their associated 95% confidence intervals (CIs) were used to estimate the strength of the association. Tests for trends were performed by adding the quartiles as continuous variables into models. We also analyzed the associations between the log-transferred SUA and the odds of depression.

Restricted cubic spline (RCS) was plotted to explore whether there were the dose–response trends between serum SUA and depression risk with the R package “rms.” 4 knots were defined at 25, 50, 75, and 95% percentiles of serum SUA, and reference value was located at the median value. *p*-values for overall and nonlinearity were calculated using a Wald test.

Subgroup analyses were adopted stratified by sex (men vs. women), age (18–39, 40–59, vs. ≥60 years), races (Hispanic, non-Hispanic White, non-Hispanic Black, vs. other races), an education level (less than high school, high school graduate, vs. more than high school), BMI (<30 vs. ≥30 kg/m^2^), history of hypertension (no vs. yes), history of diabetes (no vs. yes) and history of CVD (no vs. yes).

R software (version 4.2.3) were used to conduct all statistical analyses. *p*-values were two-sided, and statistical significance was defined as *p* < 0.05.

## 3 Results

### 3.1 Baseline characteristics

As shown in Table 1, 23,059 participants with serum SUA classified by quartile were included in the present study. The mean age of the included participants is 47.0 ± 18.7 years, with 55.1% of women. Overall, 3,426 (14.9%) participants had prevalent depression. Participants were categorized into quartiles based on the distribution of serum SUA, and the higher SUA quartiles had a relatively lower prevalence of depression compared to the lower quartiles (quartile 1: 16.2%; quartile 2: 14.8%; quartile 3: 14.5%; quartile 4: 13.9%, *p* = 0.004). Participants in the quartile 4 group tend to be older, male, non-Hispanic White or Non-Hispanic Black, receiving lower education, having higher poverty income ratio, obese, current or former smoker, moderate or heavy smoker, and having history of hypertension, diabetes, and CVD, as well as higher levels of glycated hemoglobin, TG, TC and creatinnine, and lower eGFR (all *p* < 0.05).

**Table 1:** General characteristics of the participants according to serum uric acid level within 2005–2018 NHANES survey.

Variables	Overall	Quartiles of serum uric acid, mg/dL				
		Q1	Q2	Q3	Q4	*P*
Sample size, n (%)	23,059	5,759	5,541	5,701	6,058	–
Log serum uric acid, mg/dL [mean (SD)]	1.7 (0.3)	1.3 (0.2)	1.6 (0.1)	1.7 (0.0)	2.0 (0.1)	<0.001
Depression, n (%)						0.004
No	19,633 (85.1)	4,824 (83.8)	4,720 (85.2)	4,876 (85.5)	5,213 (86.1)	
Yes	3,426 (14.9)	935 (16.2)	821 (14.8)	825 (14.5)	845 (13.9)	
Age, years [mean (SD)]	47.0 (18.7)	43.2 (17.8)	46.3 (18.6)	48.0 (18.8)	50.1 (19.0)	<0.001
Sex, n (%)						<0.001
Men	10,355 (44.9)	862 (15.0)	1961 (35.4)	3,214 (56.4)	4,318 (71.3)	
Women	12,704 (55.1)	4,897 (85.0)	3,580 (64.6)	2,487 (43.6)	1740 (28.7)	
Races, n (%)						<0.001
Hispanic	5,931 (25.7)	1793 (31.1)	1,512 (27.3)	1,424 (25.0)	1,202 (19.8)	
Non-Hispanic White	10,123 (43.9)	2,339 (40.6)	2,411 (43.5)	2,562 (44.9)	2,811 (46.4)	
Non-Hispanic Black	4,694 (20.4)	1,088 (18.9)	1,083 (19.5)	1,130 (19.8)	1,393 (23.0)	
Other races	2,311 (10.0)	539 (9.4)	535 (9.7)	585 (10.3)	652 (10.8)	
Education level, n (%)						<0.001
Below high school	3,779 (16.4)	976 (16.9)	927 (16.7)	932 (16.3)	944 (15.6)	
High school	7,575 (32.9)	1761 (30.6)	1781 (32.1)	1969 (34.5)	2064 (34.1)	
Above high school	11,705 (50.8)	3,022 (52.5)	2,833 (51.1)	2,800 (49.1)	3,050 (50.3)	
Family PIR, n (%)						<0.001
< 1	6,195 (26.9)	1,668 (29.0)	1,460 (26.3)	1,525 (26.7)	1,542 (25.5)	
1 ~ 3	9,484 (41.1)	2,280 (39.6)	2,254 (40.7)	2,343 (41.1)	2,607 (43.0)	
> 3	7,380 (32.0)	1811 (31.4)	1827 (33.0)	1833 (32.2)	1909 (31.5)	
BMI, kg/m^2^ [mean (SD)]	29.4 (7.3)	26.9 (6.3)	28.8 (7.0)	30.0 (7.2)	31.9 (7.8)	<0.001
Smoking status, n (%)						<0.001
Never	12,670 (54.9)	3,565 (61.9)	3,151 (56.9)	2,975 (52.2)	2,979 (49.2)	
Current	5,048 (21.9)	1,185 (20.6)	1,218 (22.0)	1,367 (24.0)	1,278 (21.1)	
Former	5,341 (23.2)	1,009 (17.5)	1,172 (21.2)	1,359 (23.8)	1801 (29.7)	
Drinking status, n (%)						<0.001
Never	16,915 (73.4)	4,513 (78.4)	4,183 (75.5)	4,073 (71.4)	4,146 (68.4)	
Moderate	3,070 (13.3)	650 (11.3)	676 (12.2)	842 (14.8)	902 (14.9)	
Heavy	3,074 (13.3)	596 (10.3)	682 (12.3)	786 (13.8)	1,010 (16.7)	
History of hypertension, n (%)						<0.001
No	11,243 (48.8)	3,654 (63.4)	2,993 (54.0)	2,574 (45.1)	2022 (33.4)	
Yes	11,816 (51.2)	2,105 (36.6)	2,548 (46.0)	3,127 (54.9)	4,036 (66.6)	
History of diabetes, n (%)						<0.001
No	19,277 (83.6)	5,066 (88.0)	4,726 (85.3)	4,716 (82.7)	4,769 (78.7)	
Yes	3,782 (16.4)	693 (12.0)	815 (14.7)	985 (17.3)	1,289 (21.3)	
History of CVD, n (%)						<0.001
No	22,330 (96.8)	5,665 (98.4)	5,399 (97.4)	5,531 (97.0)	5,735 (94.7)	
Yes	729 (3.2)	94 (1.6)	142 (2.6)	170 (3.0)	323 (5.3)	
Glycated hemoglobin, % [mean (SD)]	5.7 (1.1)	5.7 (1.3)	5.7 (1.1)	5.7 (1.0)	5.8 (1.0)	<0.001
TG [mean (SD)]	153.9 (132.7)	127.6 (136.4)	142.6 (117.2)	159.3 (124.1)	184.2 (143.5)	<0.001
TC [mean (SD])	191.7 (42.4)	189.1 (41.8)	191.0 (42.3)	191.8 (41.9)	194.7 (43.3)	<0.001
eGFR, mL/min per 1.73 m^2^ [mean (SD)]	96.2 (23.8)	104.9 (20.3)	98.3 (21.4)	94.5 (22.6)	86.0 (26.5)	<0.001
Serum creatinine, μmol/L [mean (SD)]	79.0 (39.6)	65.9 (34.0)	74.2 (35.9)	80.9 (34.0)	94.0 (46.7)	<0.001

Mean ± SD for normally distributed continuous variables and n (%) for categorical variables. NHANES, National Health and Nutrition Examination Survey; Q, quintile; SD, standard deviation; PIR, ratio of family income to poverty; BMI, body mass index; HPL, hyperlipidemia; HBP, hypertension; DM, diabetes mellitus; CVD, cardiovascular disease; TG, triglycerides; TC, total cholesterol; HDL, high-density lipoprotein.

### 3.2 Association between serum SUA and depression

Table 2 shows the results of the logistic regression models assessing the association between serum SUA and the risk of depression. Compared to the reference level (Q1), the association between serum SUA and depression was significantly negative in the unadjusted model [OR = 0.90 (95% CI: 0.81, 0.99) for Q2, OR = 0.87 (95% CI: 0.79, 0.97) for Q3, OR = 0.84 (95% CI: 0.76, 0.93)], and significant linear trend was found at P for trend <0.001. This association was persisted after adjusting for covariates with age, sex and race (P for trend <0.001), and the OR of quartile 4 was 0.80 (95% CI: 0.71, 0.92). Meanwhile, this association was consistent when adjusted for other covariates, and the OR of quartile 4 was 0.76 (95% CI: 0.67, 0.86). For serum SUA as continuous variables, all these three models demonstrated a strong negative association between serum SUA and depression, and the ORs and their CIs for 1 unit of log transferred serum SUA increase were 0.79 (0.69, 0.90) in model 1, 0.73 (0.62, 0.85) in model 2, and 0.68 (0.58, 0.79) in model 3.

**Table 2:** The association between serum uric acid and the odds of depression.

	Unadjusted model		Minimum adjusted model^†^		Fully adjusted model^‡^	
	OR (95%CI)	*P*	OR (95%CI)	*P*	OR (95%CI)	*P*
Log (serum uric acid)	0.79 (0.69, 0.90)	**<0.001**	1.03 (0.89, 1.19)	0.709	0.68 (0.58, 0.79)	**<0.001**
Quintiles of serum uric acid						
Q1	Ref.		Ref.		Ref.	
Q2	0.90 (0.81, 0.99)	**0.037**	0.96 (0.87, 1.06)	0.441	0.87 (0.79, 0.97)	**0.015**
Q3	0.87 (0.79, 0.97)	**0.009**	1.01 (0.90, 1.12)	0.909	0.82 (0.74, 0.92)	**<0.001**
Q4	0.84 (0.76, 0.93)	**<0.001**	1.02 (0.91, 1.14)	0.738	0.76 (0.67, 0.86)	**<0.001**
*P* for trend		**<0.001**		0.589		**<0.001**

Serum uric acid concentration (mg/dL) was log-transferred because of a skewed distribution. ^†^Adjusted for age (continuous), sex (men vs. women), and race (categorical). ^‡^Adjusted for variables in model 1 plus smoking status (categorical), drinking status (categorical), BMI (continuous), education (categorical), economic status (categorical), TC (quintile), TG (quintile), glycated hemoglobin (continuous), creatinine (continuous), eGFR (continuous) and personal history (yes vs. no) of hypertension, diabetes, and CVD. OR: odds ratio; CI: confidence interval; BMI, body mass index; HDL, high-density lipoprotein; HPL, hyperlipidemia; HBP, hypertension; DM, diabetes mellitus; CVD, cardiovascular disease; TG, triglycerides; TC, total cholesterol. The bold values represent a *p*-value < 0.05.

**Figure 2:**
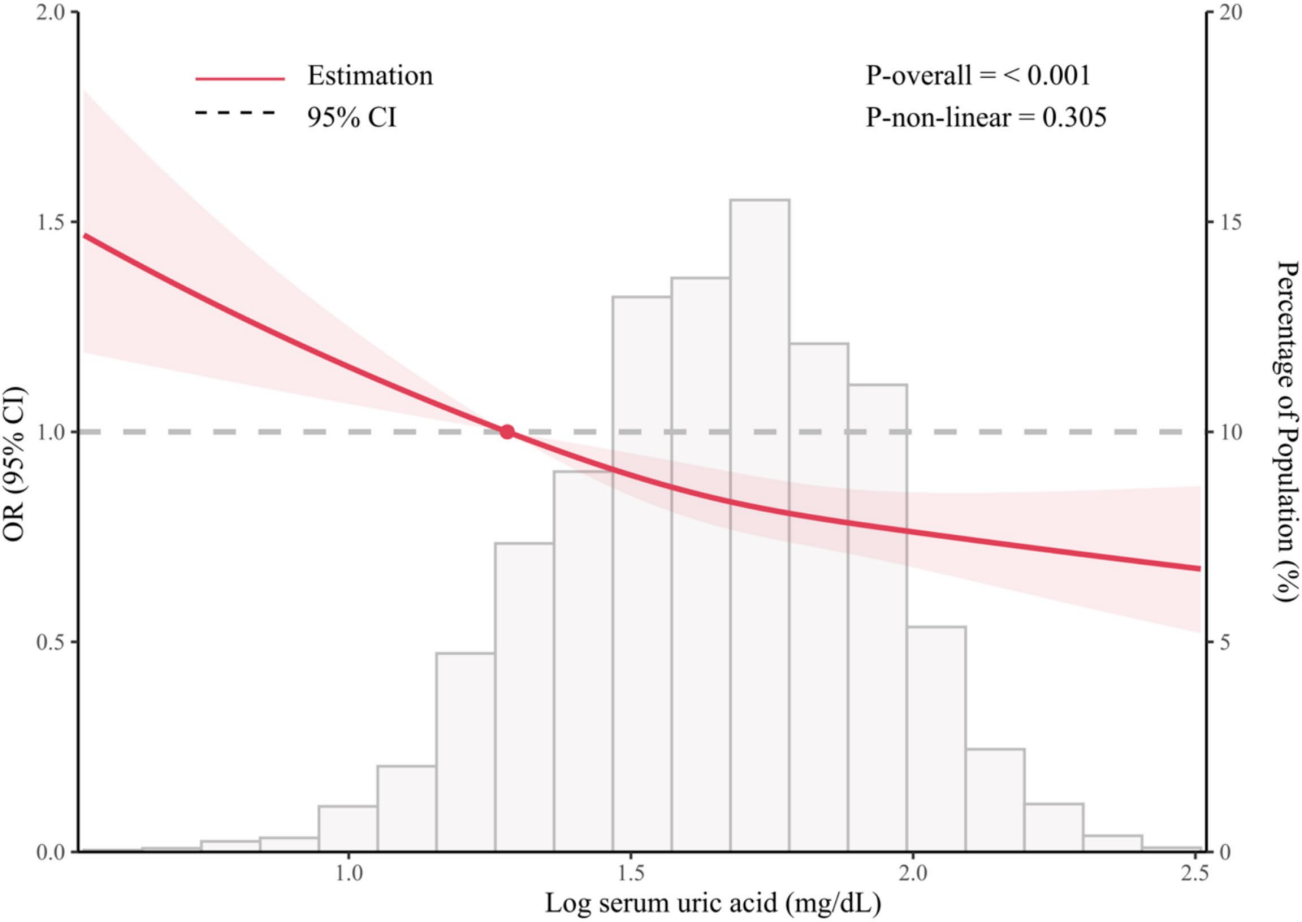
The association between serum SUA and prevent depression.

As shown in Figure 2, the restricted cubic spline model showed that significant overall trends were found between serum SUA and prevent depression (P for overall <0.001). However, non-linear association was not detected in this association (P for non-linear = 0.305), which demonstrated that threshold value of serum SUA for depression needs further to research.

### 3.3 Subgroup analyses

**Figure 3:**
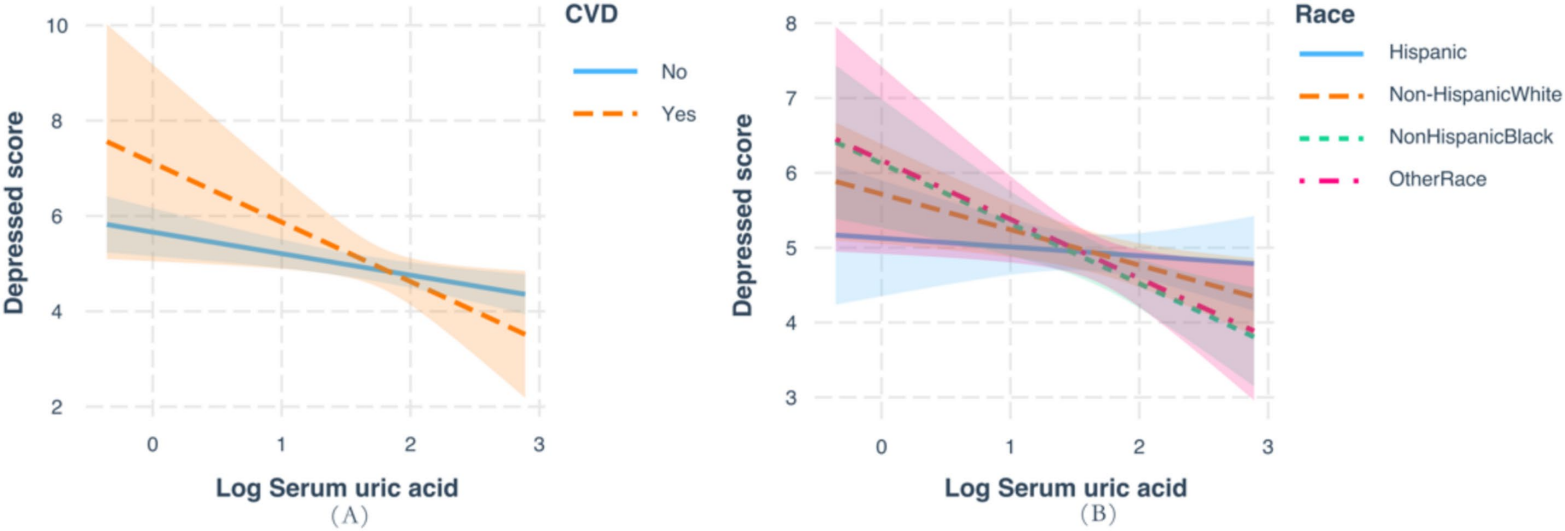
Analysis of the interaction between serum uric acid and the prevalence of depression based on race and history of cardiovascular disease.

As shown in Table 3 and Figure 3, subgroup analysis and interaction tests were conducted by sex, age, races, an education level, BMI, history of hypertension, diabetes, and CVD to examine whether there any modification effects on the association between serum and the prevalence of depression. Significant interactions were detected for races (*P* for interaction = 0.027) and history of CVD (*P* for interaction = 0.005), and marginal interactions were detected among age strata (*P* for interaction = 0.051). Noteworthy inverse effects were observed among individuals with older age (OR: 0.94 for 18–39 years, 0.87 for 40–59 years, and 0.65 for ≥60 years) and Non-Hispanic White and other races (OR: 0.97 for Hispanic, 0.70 for non-Hispanic White, 0.76 for non-Hispanic Black, and 0.50 for other races). Significant inverse associations were more evident among those with a history of CVD (OR: 0.40; *p* < 0.001). No significant interactions were observed for other factors, such as sex, BMI, and history of diabetes and hypertension.

**Table 3:** The stratified analyses for the association between serum uric acid and odds of depression.

Subgroups	OR (95%CI)				*P* for trend	*P*-interaction
	Q1	Q2	Q3	Q4		
Sex						0.905
Men	Ref.	0.94 (0.74, 1.19)	0.86 (0.69, 1.09)	0.78 (0.62, 0.98)	0.009	
Women	Ref.	0.86 (0.76, 0.97)	0.82 (0.71, 0.95)	0.78 (0.65, 0.92)	0.001	
Age group, years						0.051
18–39	Ref.	0.87 (0.73, 1.04)	0.89 (0.73, 1.09)	0.94 (0.74, 1.18)	0.520	
40–59	Ref.	0.98 (0.82, 1.18)	0.95 (0.78, 1.15)	0.87 (0.70, 1.08)	0.202	
≥ 60	Ref.	0.78 (0.63, 0.97)	0.73 (0.59, 0.90)	0.65 (0.52, 0.80)	<0.001	
Races						0.027
Hispanic	Ref.	0.90 (0.74, 1.10)	0.94 (0.76, 1.16)	0.97 (0.76, 1.23)	0.809	
Non-Hispanic White	Ref.	0.86 (0.73, 1.02)	0.75 (0.63, 0.90)	0.70 (0.58, 0.85)	<0.001	
Non-Hispanic Black	Ref.	0.84 (0.66, 1.07)	0.82 (0.64, 1.06)	0.76 (0.58, 0.99)	0.053	
Other Races	Ref.	0.87 (0.60, 1.28)	0.76 (0.51, 1.14)	0.50 (0.32, 0.79)	0.003	
BMI groups, kg/m^2^						0.284
< 30	Ref.	0.83 (0.72, 0.95)	0.80 (0.68, 0.93)	0.73 (0.61, 0.87)	0.000	
≥ 30	Ref.	0.97 (0.82, 1.16)	0.91 (0.76, 1.08)	0.85 (0.71, 1.02)	0.046	
eGFR, mL/min per 1.73 m^2^						0.248
< 60	Ref.	1.60 (0.35, 7.33)	1.24 (0.24, 6.51)	0.74 (0.12, 4.40)	0.250	
60 ~ 90	Ref.	1.13 (0.57, 2.24)	1.26 (0.61, 2.63)	0.99 (0.47, 2.10)	0.956	
> 90	Ref.	0.94 (0.67, 1.33)	1.00 (0.56, 1.76)	0.85 (0.42, 1.72)	0.688	
DM						0.119
No	Ref.	0.88 (0.78, 0.99)	0.77 (0.68, 0.88)	0.74 (0.64, 0.85)	0.000	
Yes	Ref.	0.88 (0.68, 1.16)	1.05 (0.81, 1.36)	0.85 (0.65, 1.10)	0.379	
Hypertension						0.823
No	Ref.	0.84 (0.72, 0.98)	0.85 (0.71, 1.01)	0.72 (0.58, 0.89)	0.004	
Yes	Ref.	0.92 (0.78, 1.07)	0.83 (0.71, 0.97)	0.80 (0.68, 0.94)	0.003	
CVD						**0.005**
No	Ref.	0.88 (0.79, 0.98)	0.83 (0.74, 0.93)	0.78 (0.69, 0.88)	<0.001	
Yes	Ref.	0.69 (0.34, 1.40)	0.64 (0.32, 1.28)	0.40 (0.21, 0.79)	0.006	

The stratified analyses were conducted in the full-adjusted model. OR: odds ratio; CI: confidence interval; BMI, body mass index; DM, diabetes mellitus; CVD, cardiovascular disease. The bold values represent a *p*-value < 0.05.

## 4 Discussion

In this large-scale, representative, cross-sectional study, we found that a higher serum UA level was associated with a significantly decreased prevalence of depression among U.S. adults. A variety of stratified analyses revealed that this effect was modified by age, race and pre-existing comorbidities (e.g., CVD), with negative associations being more pronounced among older participants, other races, and participants with a diagnosis of CVD. Consistently, our findings may lead to clinical recommendations to measure the circulating UA concentration among patients with depression.

Our data revealed that participants with higher quintiles of serum UA (Q2-Q4) had lower odds of depression compared with those with the lowest quintile (Q1), with the log-transformed serum UA consistently showing a negative association with depression. The present study was consistent with previous studies (16–19, 28). In a study involving 1,543 patients with depression and 1,515 healthy controls from a mental health center in southwestern China, it was found that serum UA levels were lower in depressed patients compared to controls (16). A previous study in Denmark suggested that higher plasma concentration of UA was related to lower depression hospitalization (OR = 0.57, 95%CI 0.49–0.65) and anti-depressant medication used (OR = 0.77, 95%CI 0.73–0.81) (17). Subsequently, a Korean study indicated that a log-transformed serum UA remained inversely associated with depressive symptoms after adjusting for covariates [incidence rate ratio (IRR) = 0.64, 95%CI 0.45–0.92] (19). A Chinese study consistently reported the inverse association between serum UA level and depression prevalence (OR = 0.57, 95%CI 0.41–0.81) (18). Individual with higher serum UA levels had a lower risk of depressive symptoms (OR = 0.921; 95%CI: 0.886–0.957) in East Asian populations (29). Consistently, there were negative associations of serum UA levels with depressive symptoms in other studies (30, 31), in peritoneal dialysis patients (32), as well as the positive association with better baseline cognition and less subsequent cognitive decline among Chinese adults (33). However, there are discrepancies in the findings between studies. Study found that high serum UA was associated with depression, especially in bipolar disorder (16). In addition, a study from Korea reported opposing associations between SUA and depressive symptoms depending on the presence of low-grade inflammation (19), suggesting that inflammation could modify the relationship between SUA and depression. Furthermore, a Chinese study of middle-aged and elderly participants found a negative association between SUA and depression in men but not in women (18). These findings indicate that some of these inconsistencies could arise from the varying health conditions and clinical characteristics of participants across studies.

Although the pathological mechanism of depression is still unclear, oxidative stress may be a causal factor, with upstream and downstream mechanisms being involved in the pathophysiology of depression (34). A variety of downstream biological effects are triggered by cytokine signaling, including impact on the neuroendocrine system, and the monoaminergic system, thus facilitating the development of “sickness behavior” such as depressed mood, fatigue, and loss of appetite (34). As the end product of purine metabolism, UA has intracellular pro-oxidant properties but exerts an antioxidant effect in human circulation (19). The antioxidant capacity of serum UA could be presented among depression patients with low-grade inflammation, and the relatively higher anti-oxidant capacity would protect them from oxidative and nitrosative stress, as well as the damage from cellular components (fatty acids, proteins, DNA, and mitochondria) (35). The neuro-inflammation caused by the upstream mechanisms would further affect serotonergic, glutamatergic, and dopaminergic circuits by changing the metabolism of tryptophan [the kynurenine pathway (KP)] (36). Notably, higher antioxidant levels owing to elevated serum UA level could block the enzyme indoleamine dioxygenase, lead to lower kynurenine and higher serotonin levels, remove reactive oxygen species (ROS) and free radicals, and finally reduce the depression risk (35, 37). Furthermore, the cause of depression has been linked to impaired neuroplasticity (38), dysregulation of the hypothalamus-pituitary–adrenal (HPA) axis, and alteration of the continuous production of adult-generated neurons in the dentate gyrus of the hippocampus (36). Considering together with oxidative stress, the purine metabolism pathway may be one additional mechanism, and the purinergic metabolism could affect neurotransmitter systems and hormonal pathways in the HPA axis, thus being involved in the pathophysiology of mood disorders (39). Given the complexity of the above mechanisms, more investigations are needed to validate the associations between oxidative stress, inflammation, and depressive symptoms, and to identify additional biological markers for depression.

After adjusting for all covariates, most of the stratified analyses demonstrated the robustness of our findings, except for the modification effects of age, race and the history of CVD. We observed that age significantly influenced the protective effect of serum UA on depression (p interaction = 0.51), with older individuals showing a more pronounced association. This finding suggests that the relationship between SUA levels and depression may strengthen with increasing age. As individuals age increased, there is a notable increase in inflammatory responses and oxidative stress levels, which are linked to a higher prevalence of chronic diseases, including depression (40). Thus, more pronounced benefits of elevated SUA levels in older adults by acting on age-related increased in inflammation and oxidative stress, potentially leading to more significant benefits in reducing depression risk.

The inverse association between serum UA level and depression was more pronounced among those in the “other ethnicities” category (that included Americans of Asian, Pacific Island, and Native American heritage), underlying the potential heterogeneity due to race. Being partly in line with our findings, recent NHANES-based results also noticed higher serum urate concentrations and gout prevalence in US Asian Adults, compared with white individuals, indicating potential genetic risk factors (41) Concerning to the potential differences from other races, further studies with strong evidence are needed to confirm this association in the “other race” category.

We also found that the history of CVD acted as a potential effect modifier of the serum UA-depression relationship, with the negative association tended to occur in those with pre-existing CVD. This notion might also be supported by the shared pathological mechanisms between CVD and purine metabolism. Literature has suggested the relationships between elevated serum UA and a series of CVDs, including hypertension (42), atrial fibrillation (43), silent myocardial infarction (44), as well as the association of hyperuricemia with atherosclerotic CVDs (45). The causative roles of elevated serum UA in vascular damage and remodeling have been reported by prior experimental studies (46, 47). On the one hand, xanthine oxidoreductases (XOR), such as xanthine oxidase (XO) and xanthine dehydrogenase (XDH), catalyze the conversion of hypoxanthine to xanthine, and xanthine to UA (48). On the other hand, plasma XOR activity associated with higher risk of CVDs has been demonstrated in a Japanese population-based cohort study (49). Further studies are needed to clarify the modification effect of CVD on these relationships.

Our results have several obvious advantages. The present study was conducted using a large sample with a total of 23,059 participants from the NHANES 2013–2018 survey, by adopting multi-layer random sampling with the results generalized to the entire U.S. population. Despite the strengths of our study, including its large, nationally representative sample and rigorous statistical analyses, several limitations must be acknowledged. The cross-sectional design of the study precludes causal inference, leaving the directionality of the serum uric acid (UA)-depression relationship uncertain. Longitudinal or experimental studies are needed to clarify whether elevated serum UA levels are a cause or consequence of depression. Additionally, while we adjusted for numerous potential confounders, there remains the possibility of residual confounding due to unmeasured factors such as diet, physical activity, genetic predispositions and health problems such as Alzheimer’s disease or dementia. Moreover, the inclusion of only U.S. adults limits the generalizability of our findings to other populations with different genetic, cultural, or environmental contexts. Although we proposed potential biological mechanisms linking UA to depression, these remain speculative. Specifically, UA has been implicated in oxidative stress, inflammation, and endothelial dysfunction, all of which may contribute to depression’s pathophysiology. Future research should investigate these pathways in greater depth, examining UA’s dual role as both an antioxidant and pro-oxidant, as well as its modulation of cytokine signaling and impact on neurovascular health. A more comprehensive, multi-biomarker approach, incorporating other markers of oxidative stress, inflammation, and neuroplasticity, could provide a broader understanding of the biochemical and molecular underpinnings of the UA-depression relationship. Addressing these limitations and investigating the proposed mechanisms in future research will be crucial to enhancing the validity and applicability of our findings.

## 5 Conclusion

In a nationally representative sample of U.S. adults, the current study provides evidence to suggest that higher circulating concentrations of UA were inversely associated with the odds of depression, with the association being more pronounced among those with older ages, other races and with a history of CVD. These results highlight that adjusting the serum UA level in depression patients having circulating serum UA within the lower normal range might help restore the ability of antioxidant stress and protect brain function.

## Data Availability

The datasets presented in this study can be found in online repositories. The names of the repository/repositories and accession number(s) can be found at: https://www.cdc.gov/nchs/nhanes/index.htm.

## Footnotes

^1^ https://www.cdc.gov/nchs/nhanes/
