# Supplementary material for: The association between serum uric acid and depression among U.S. National Health and Nutrition Examination Survey
Source: Front Nutr. 2025 Apr 8;12:1517744. doi: 10.3389/fnut.2025.1517744 (PMC12011595; doi:10.3389/fnut.2025.1517744)
Supplement: Supplementary file 1 [file Table_1.docx]

**Table S1. Biochemical characteristics of the participants according to hyperuricemia status within 2005-2018 NHANES survey.**

| **Variables** | **Overall** | **No hyperuricemia** | **Hyperuricemia** | **p** |
| --- | --- | --- | --- | --- |
| **Sample size, n (%)** | 23,059 | 18,069 | 4,990 |  |
| **Log serum uric acid, mg/dL (mean [SD])** | 1.7 (0.3) | 1.6 (0.2) | 2.0 (0.1) | <0.001 |
| **Glycated hemoglobin, % (mean [SD])** | 5.7 (1.1) | 5.7 (1.1) | 5.9 (1.0) | <0.001 |
| **TG,mg/dL (mean [SD])** | 153.9 (132.6) | 146.8 (131.8) | 179.7 (132.3) | <0.001 |
| **TC,mg/dL (mean [SD])** | 191.7 (42.4) | 190.3 (42.2) | 196.8 (42.8) | <0.001 |
| **HDL,mg/dL (mean [SD])** | 53.0 (16.1) | 53.9 (16.1) | 49.7 (15.6) | <0.001 |
| **Plasma glucose, mmol/L (mean [SD])** | 6.0 (2.0) | 6.0 (2.1) | 6.2 (1.9) | <0.001 |
| **Serum creatinine, umol/L (mean [SD])** | 79.7 (35.7) | 76.6 (35.2) | 91.8 (35.4) | <0.001 |

**Note**: Mean ± SD for normally distributed continuous variables and n (%) for categorical variables

**Abbreviations**: NHANES, National Health and Nutrition Examination Survey; SD, standard deviation; TG, triglycerides; TC, total cholesterol; HDL, high-density lipoprotein.

**Table S2. Biochemical characteristics of the participants according to depressed status within 2005-2018 NHANES survey.**

| **Variables** | **Overall** | **No depressed** | **Depressed** | **p** |
| --- | --- | --- | --- | --- |
| **Sample size, n (%)** | 23,059 | 19,633 | 3,426 |  |
| **Log serum uric acid, mg/dL (mean [SD])** | 1.7 (0.3) | 1.7 (0.3) | 1.6 (0.3) | <0.001 |
| **Glycated hemoglobin, % (mean [SD])** | 5.7 (1.1) | 5.7 (1.1) | 5.9 (1.3) | <0.001 |
| **TG,mg/dL (mean [SD])** | 153.9 (132.6) | 151.7 (130.6) | 166.3 (143.3) | <0.001 |
| **TC,mg/dL (mean [SD])** | 191.7 (42.4) | 191.3 (42.0) | 193.8 (44.6) | 0.001 |
| **Plasma glucose, mmol/L (mean [SD])** | 6.0 (2.0) | 6.0 (2.0) | 6.3 (2.4) | <0.001 |

**Note**: Mean ± SD for normally distributed continuous variables and n (%) for categorical variables

**Abbreviations**: NHANES, National Health and Nutrition Examination Survey; SD, standard deviation; TG, triglycerides; TC, total cholesterol; HDL, high-density lipoprotein.

**Table S3. The stratified analyses for the association between serum uric acid and odds of depression in elderly participants (age >60 years).**

| **Subgroups** | **OR (95%CI)** | | | | ***P* for trend** | ***P*-interaction** |
| --- | --- | --- | --- | --- | --- | --- |
|  | **Q1** | **Q2** | **Q3** | **Q4** |  |  |
| **eGFR, mL/min per 1.73 m2** |  |  |  |  |  | p= 0.409 |
| <60 | Ref. | 1.45 (0.17,12.38) | 1.13 (0.15,8.81) | 0.82 (0.09,7.40) | 0.494 |  |
| 60~90 | Ref. | 0.83 (0.35,1.96) | 0.97 (0.27,3.50) | 0.88 (0.26,2.92) | 0.908 |  |
| >90 | Ref. | 0.85 (0.24,3.06) | 1.81 (0.39,8.31) | 0.36 (0.06,2.23) | 0.497 |  |

**Notes**: The stratified analyses were conducted in the full-adjusted model.
