# Supplementary figures and images for: The association between serum uric acid and depression among U.S. National Health and Nutrition Examination Survey
Source: Front Nutr. 2025 Apr 8;12:1517744. doi: 10.3389/fnut.2025.1517744 (PMC12011595; doi:10.3389/fnut.2025.1517744)

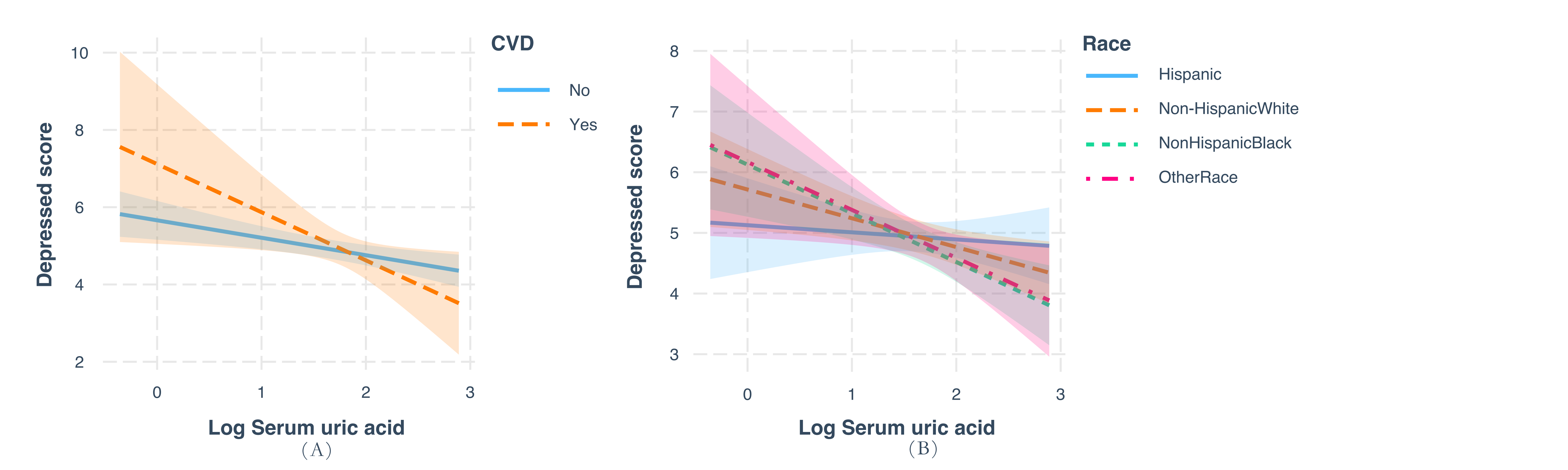

Supplement: Supplementary file 2 [file Image_1.png]

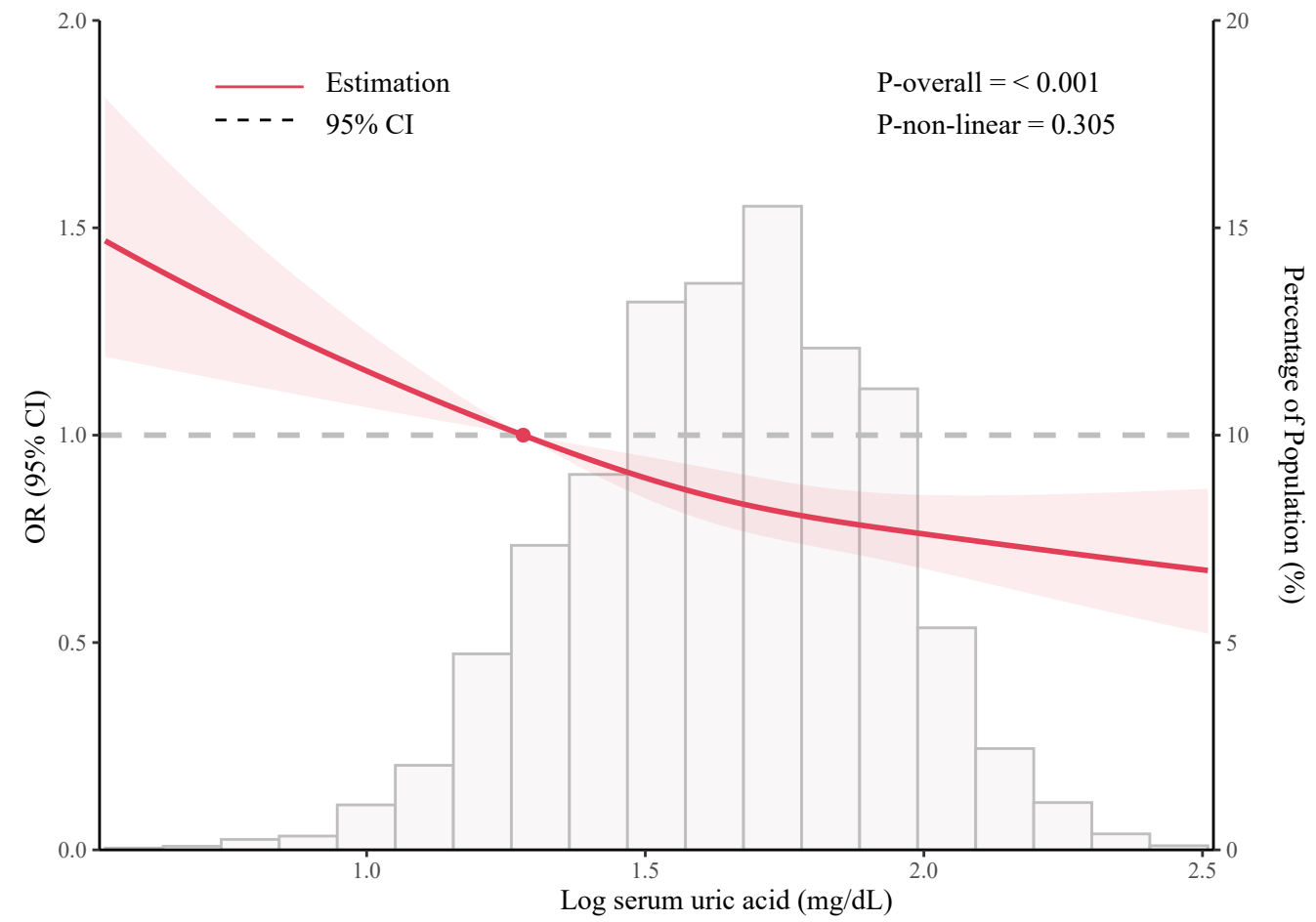

Supplement: Supplementary file 3 [file Image_2.pdf]
